# Supplementary material for: Continuous evolution of a halogenase enzyme with improved solubility and activity for sustainable bioproduction
Source: Nat Commun. 2026 Mar 24;17:4357. doi: 10.1038/s41467-026-70981-4 (PMC13171985; doi:10.1038/s41467-026-70981-4)
Supplement: Supplementary file 1 — Supplemental Information [file 41467_2026_70981_MOESM1_ESM.pdf]

Supplementary Information

Supplementary Table 1 - Plasmids used in this study

Plasmids used in this study, and the respective panels in which they were used. Full plasmid maps for every construct are available in the Supplementary data file. Selected plasmids (*) are available on Addgene under accession numbers 247093 - 247103.

Figure	Plasmids used
Fig. 1B, 1C and 1D	pAP79* + pAP49 (Biosensor circuit)
Fig. 1F	pAP79* + pAP49 (Biosensor circuit), pAP79* + pAP65* (RebH WT + Fre), pAP79* + pAP62 (MBP + Fre)
Fig. 2B, 2C	pAP79* (AP1) + pAP70* (AP2), pAP77 (empty phage genome), pAP68 (RebH phage genome)
Fig. 3B	pAP79* (AP1) + pAP70* (AP2) + MP6, pAP68 (RebH phage genome), pAP93 ($RebH_{Evo1}$ phage genome), pAP105 ($RebH_{Evo3}$ phage genome)
Fig. 3D	pAP79* (Biosensor), pAP65* (RebH WT) + pAP79*, pAP94 ($RebH_{Evo1}$) + pAP79*, pAP102 ($RebH_{Evo2}$) + pAP79*, pAP104 ($RebH_{Evo3}$) + pAP79*, pAP113* ($RebH_{Evo4}$) + pAP79*
Fig. 4A, 4B	pAP65* (RebH WT), pAP113* ($RebH_{Evo4}$)
Fig. 4C	pAP108 (RebH WT under T7 promoter), pAP109 ($RebH_{Evo4}$ under T7 promoter)
Fig. 4D	pAP65* (RebH WT) + pAP79*, pAP54* (Fre-L3-RebH WT) + pAP79*, pAP113* ($RebH_{Evo4}$) + pAP79*, pAP114* (Fre-L3-$RebH_{Evo4}$) + pAP79*
Fig. 4E	pAP114* (Fre-L3-$RebH_{Evo4}$ GFP*1TAG) + pAP80*
Fig. 5A	pAP65* (RebH WT) or pAP113* ($RebH_{Evo4}$), pJB21x05* (RgnTDC)
Fig. 5B	pJB23x02 (proK-lacO $RebH_{Evo4}$, Fre)
Fig. 5C, 5D	pJB15x02* (Enterocin RJ-11 WT), pJB15x06v1, 2*, 3 (Enterocin RJ-11 W12, 30, 38 TAG)
Fig. 5F, 5G	pJB18x02v1* (T7-lacO SUMO-Enterocin RJ-11 W30 TAG), pAP80*
Supplementary Fig. 1A	pAP58 (ChPhe tRNA) + pAP49, pAP63 ($tRNA_{3C11}$) + pAP49
Supplementary Fig. 1B	pAP63 ($tRNA_{3C11}$) + pAP49, pAP79* (*S333C + $tRNA_{3C11}$ tRNA)
Supplementary Fig. 2B, 2D, 2E	pAP65* (RebH WT) + pAP79*, pAP94 ($RebH_{Evo1}$) + pAP79*, pAP102 ($RebH_{Evo2}$) + pAP79*, pAP104 ($RebH_{Evo3}$) + pAP79*, pAP113* ($RebH_{Evo4}$) + pAP79*
Supplementary Fig. 3 and Supplementary Fig. 4	pAP65* (RebH WT), pAP113* ($RebH_{Evo4}$)
Supplementary Fig. 5A	pASx48a (pET-28a_MBP-RebF), pASx49 (pET-28a_$RebH_{WT}$), pASx50 (pET-28a_$RebH_{Evo4}$)
Supplementary Fig. 5B and Supplementary Fig. 6A	pASx49 (pET-28a_$RebH_{WT}$), pASx50 (pET-28a_$RebH_{Evo4}$)
Supplementary Fig. 7A	pAP65* (RebH WT) + pAP79*, pAP113* ($RebH_{Evo4}$) + pAP79*, pJB24x01 (His-Thrombin-RebH 3-LR) + pAP79*, pJB24x02 (His-Thrombin-RebH 3-LSR) + pAP79*, pJB24x04 (His-Thrombin-RebH WT) + pAP79*, pJB24x05 (RebH 3-LR) + pAP79*, pJB24x06 (RebH 3-LSR) + pAP79*, pAP49 (No RebH) + pAP79*
Supplementary Fig. 8A	pAP118 (WT GFP), pAP54* (Fre-L3-RebH WT) + pAP80*, pAP114* (Fre-L3-$RebH_{Evo4}$ GFP*1TAG) + pAP80*, pAP119 (Fre-L3-$RebH_{Evo4}$ GFP*3TAG) + pAP80*
Supplementary Fig. 8B	pAP114* (Fre-L3-$RebH_{Evo4}$ GFP*1TAG) + pAP80*
Supplementary Fig. 9A	pJB15x02* (Enterocin RJ-11 WT), pJB15x06v1, 2*, 3 (Enterocin RJ-11 W12, 30, 38 TAG)
Supplementary Fig. 9B	pJB18x02v1* (T7-lacO SUMO-Enterocin RJ-11 W30 TAG), pAP80*

Supplementary Table 2 - Fold change tested RebH variants

Fold change and GFP/OD_{600} data of tested RebH variants (using DH10B cells) from Supplementary Fig. 2B, 2D and 2E.

RebH variant tested	Average GFP/OD readings	Fold-change (Versus RebH WT)	Fold-change versus starting point (best mutant only)
$RebH_{WT}$	36149.34	1	
$RebH_{WT}$ + V256I + T385I	77041.99	2.131214	
$RebH_{WT}$ M430L + V256I + T385I	69103.07	1.9115997	
$RebH_{WT}$ M430L +V256I +T385I +T348A	94695.285	2.6195577	2.6195577
$RebH_{WT}$	39412.835	1	
$RebH_{evo1}$	124818.8	3.1669581	
$RebH_{evo1}$ + A16S	161739.575	4.1037285	
$RebH_{evo1}$ + A16S + A50T	74886.655	1.9000575	
$RebH_{evo1}$ + A16S + T494R	184907.7	4.6915605	
$RebH_{evo1}$ + A16S + D101N	180929.167	4.5906153	
$RebH_{evo1}$ + A16S + T494R + N326K	199031.1	5.0499056	1.5945602
$RebH_{WT}$	34033.995	1	
$RebH_{evo1}$	109167.9	3.2076134	
$RebH_{evo2}$	143470.225	4.2154976	
$RebH_{evo2}$+D101N	165350	4.8583776	
$RebH_{evo2}$+A32V	182125.625	5.3512855	
$RebH_{evo2}$+G102S	155949.05	4.5821552	
$RebH_{evo2}$+P138S	114911.625	3.3763777	
$RebH_{evo2}$+R509C	165639.275	4.8668772	
$RebH_{evo2}$+Q494K	162898.425	4.7863445	
$RebH_{evo2}$ + D101N + A32V+ Q494K	201539.15	5.9217012	1.4047454
$RebH_{evo2}$ + D101N + A32V+ Q494K + R509C	192769.375	5.6640243	
$RebH_{evo2}$ + D101N + A32V+ Q494K + R509C + E186K + L233M	178405.6	5.2419823	
$RebH_{evo2}$ + D101N + A32V+ Q494K + R509C + L233M + G504S	170182.575	5.0003702	
$RebH_{WT}$	21364.69	1	
$RebH_{evo3}$	104125.033	4.8736973	
$RebH_{evo3}$+P416S+Q323H	114160.7	5.3434288	1.0963809
$RebH_{evo3}$+P416S+V481I	92660.6867	4.3370948	
$RebH_{evo3}$+P416S+V481I+Q323H	108097.7	5.0596428	
$RebH_{evo3}$+P416S+Q323H+R217C	85396.4667	3.9970842	
$RebH_{evo3}$+P416S+A445T+R217C	61351.6233	2.8716364	
$RebH_{evo3}$+P416S+R217C+D483N	78694.2233	3.6833777	
$RebH_{evo3}$+P416S+A445T	66987.4367	3.1354275	

Supplementary Figures

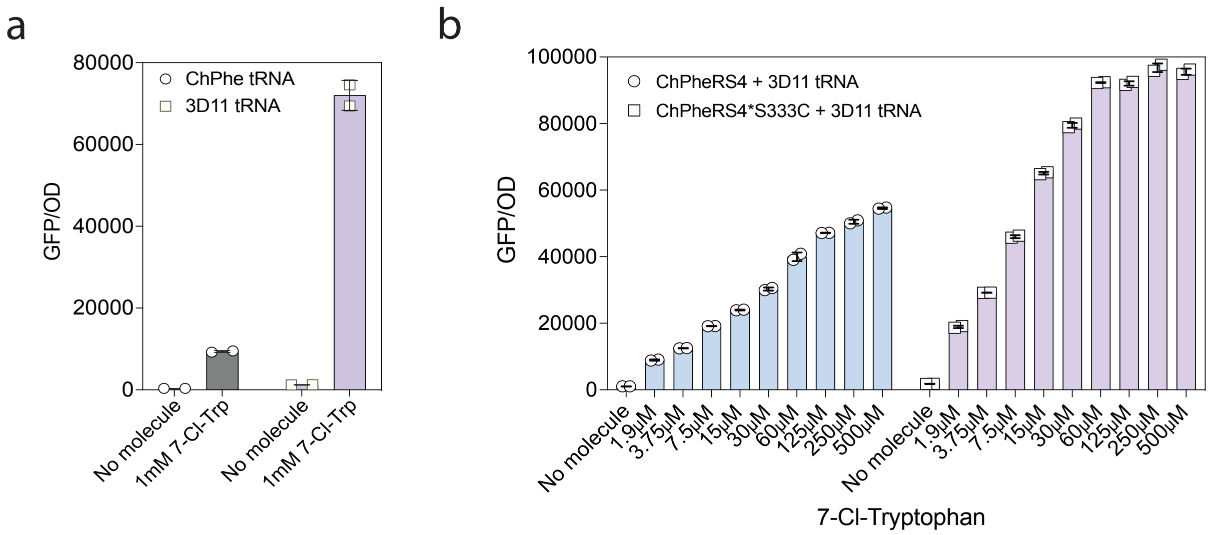

Supplementary Fig. 1: Biosensor tRNA and aaRS tuning

1a. Amber suppression of sfGFP using either the original ChPheRS4 tRNA, or the improved 3D11 tRNA, in the presence of absence of 7-Cl-Trp. Error bars show mean and standard deviation between 2 biological replicates.

1b. Amber suppression of sfGFP using either the original ChPheRS4, or an improved mutant ChPheRS4*S333C, in the presence or absence of 7-Cl-Trp. Error bars show mean and standard deviation between 2 biological replicates.
Source data for this figure is available in the Source Data file 1.

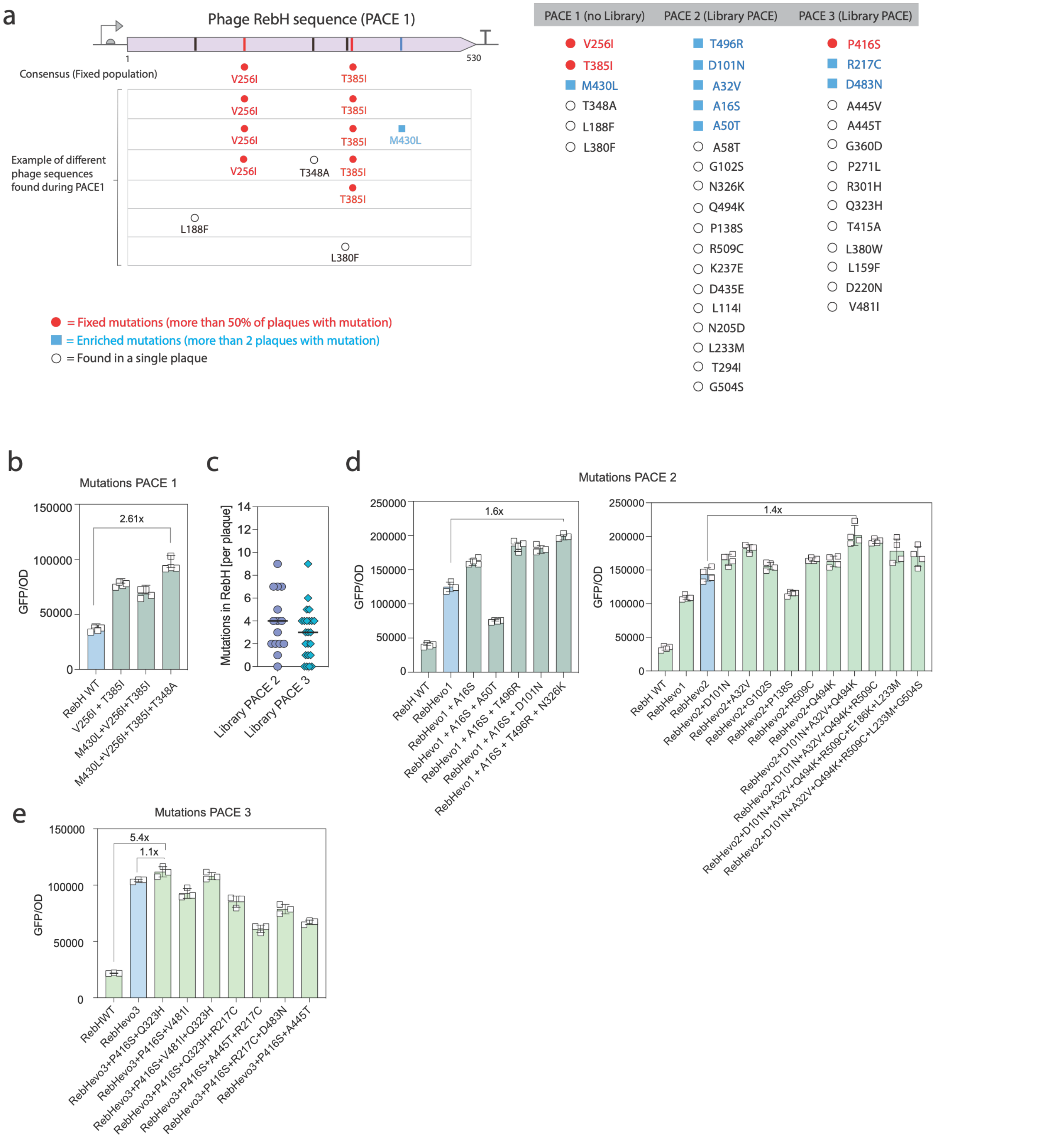

Supplementary Fig. 2 - Combination of mutations from PACE runs

2a. Results from the first PACE run, highlighting the different mutations obtained, and their abundances (on the right). On the left, list of mutations recovered on clonal phage on each run.

2b. Combination of different mutations obtained during PACE 1, using sfGFP circuit. Error bars show mean and standard deviation between 4 biological replicates.

2c. Estimation of number of mutations per plaque in each library used for PACE 2 and PACE 3, after sequencing individual plaques. Black bar shows the average of mutations per library.

2d. Combination of different mutations obtained during PACE 2, using sfGFP circuit. Error bars show mean and standard deviation between 4 biological replicates.

2e. Combination of different mutations obtained during PACE 3, using sfGFP circuit. Error bars show mean and standard deviation between 3 biological replicates.

Source data for this figure is available in the Source Data file 1.

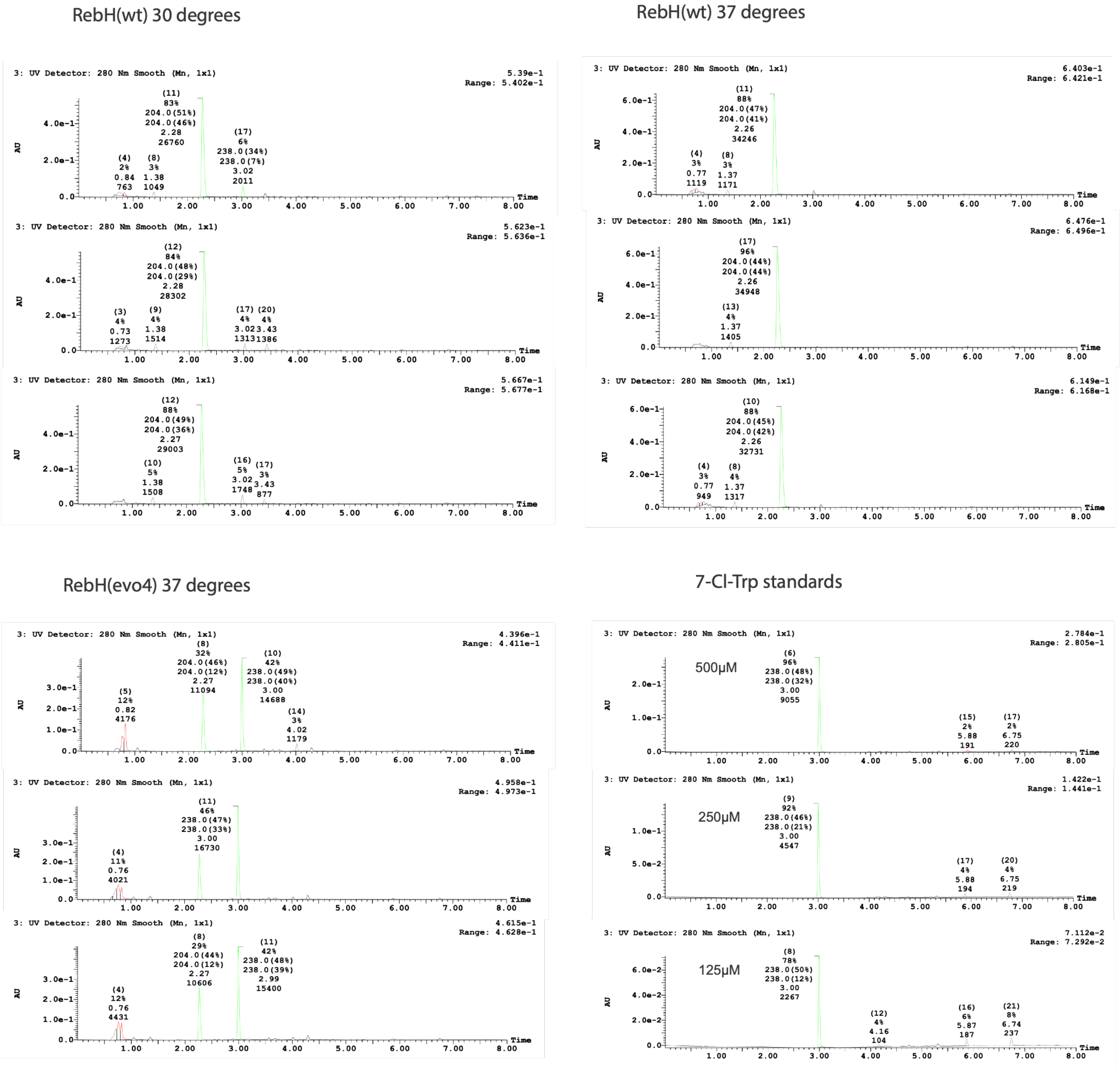

Supplementary Fig. 3 - 7-Cl-Trp HPLC raw spectra

HPLC spectra from 7-Cl-Trp production using either $RebH_{WT}$ or $RebH_{Evo4}$

RebH(wt) 30 degrees

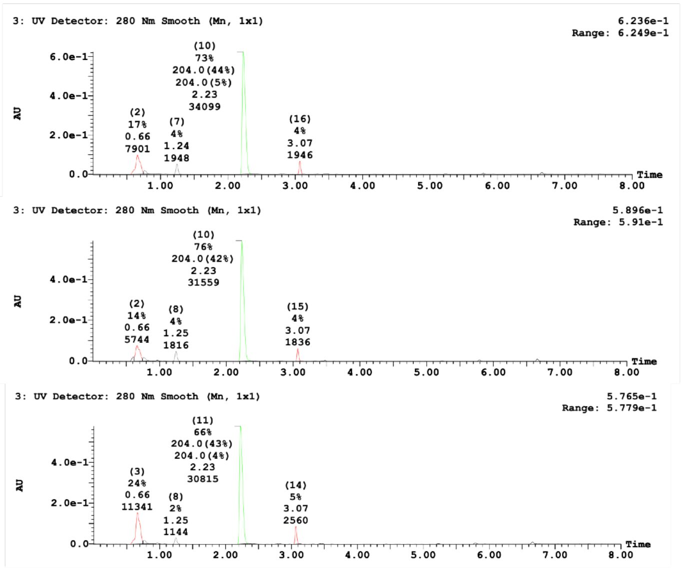

RebH(wt) 37 degrees

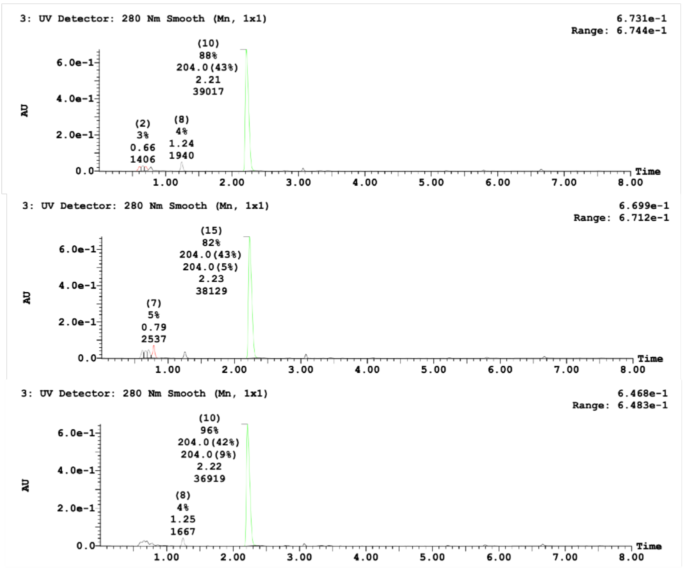

RebH(evo4) 37 degrees

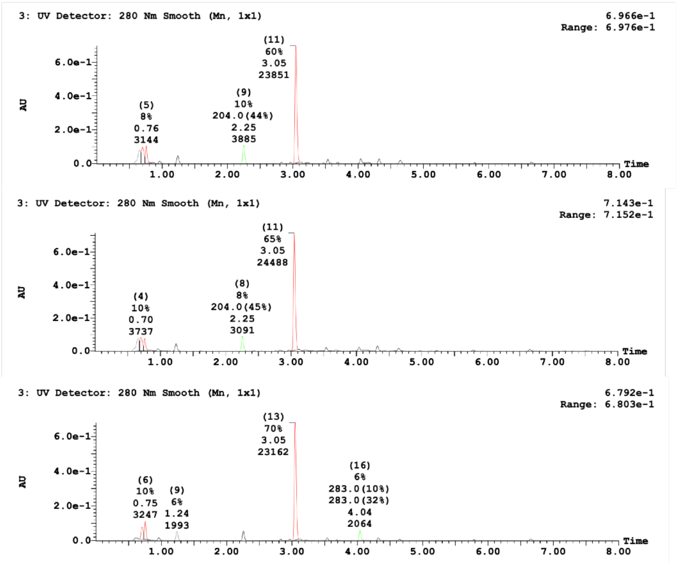

7-Br-Trp standards

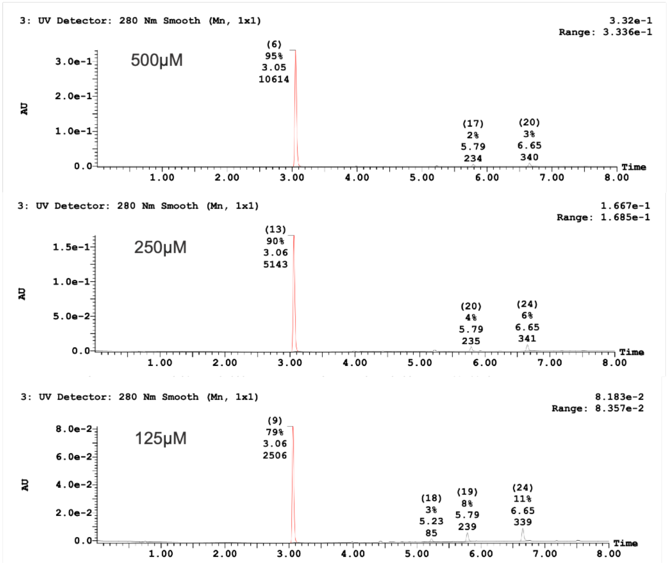

Supplementary Fig. 4 - 7-Br-Trp HPLC raw spectra

HPLC spectra from 7-Br-Trp production using either $RebH_{WT}$ or $RebH_{Evo4}$

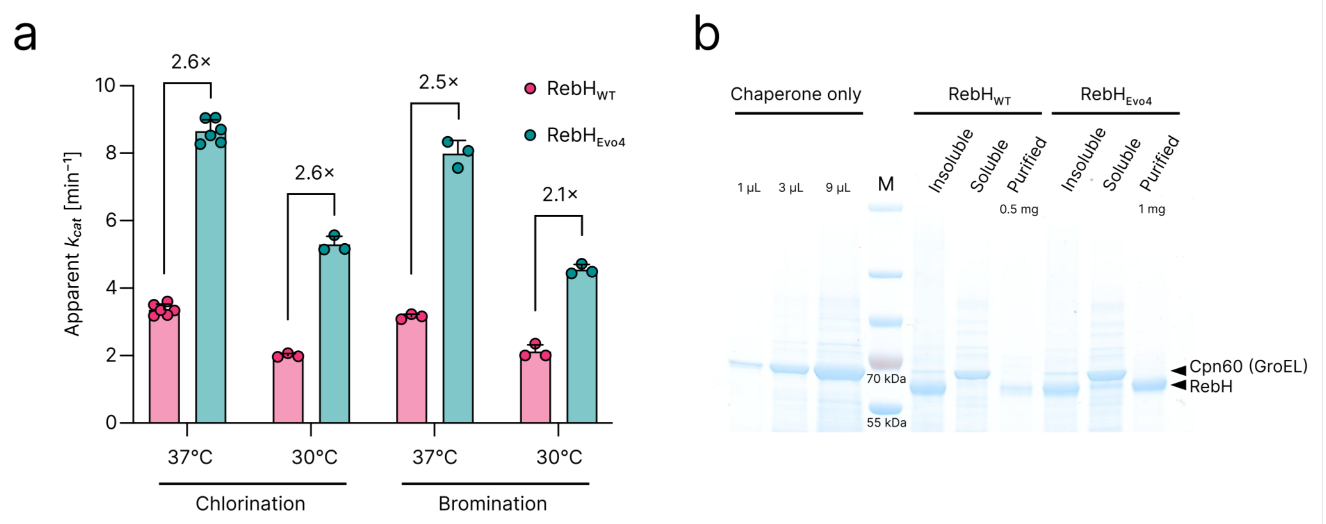

Supplementary Fig. 5 - $RebH_{Evo4}$ in vitro characterisation

5a In vitro enzyme activities of purified RebH variants. Data represents the mean initial conversion rates of enzyme reactions performed in triplicate (6 replicates for chlorination at 37°C), with HPLC-based product quantification from at least three time-points. Bars show mean and error bars show standard deviation, and fold-change is indicated by brackets.

5b SDS-PAGE confirming purification of $RebH_{WT}$ and $RebH_{Evo4}$ and successful removal of GroEL chaperone (Cpn60). The leftmost three lanes show total, uncleared lysate of untransformed ArcticExpress(DE3) cells, with a dominant band representing the Cpn60 chaperone. Similar volumes of each fraction were loaded into the gel. Note that a lower amount of purified $RebH_{WT}$ (0.5 mg) was loaded than for $RebH_{Evo4}$ (1 mg). Electrophoresis was run for an extended amount of time for optimal resolution in the 55-70 kDa range.

Source data for this figure is available in the Source Data file 1.

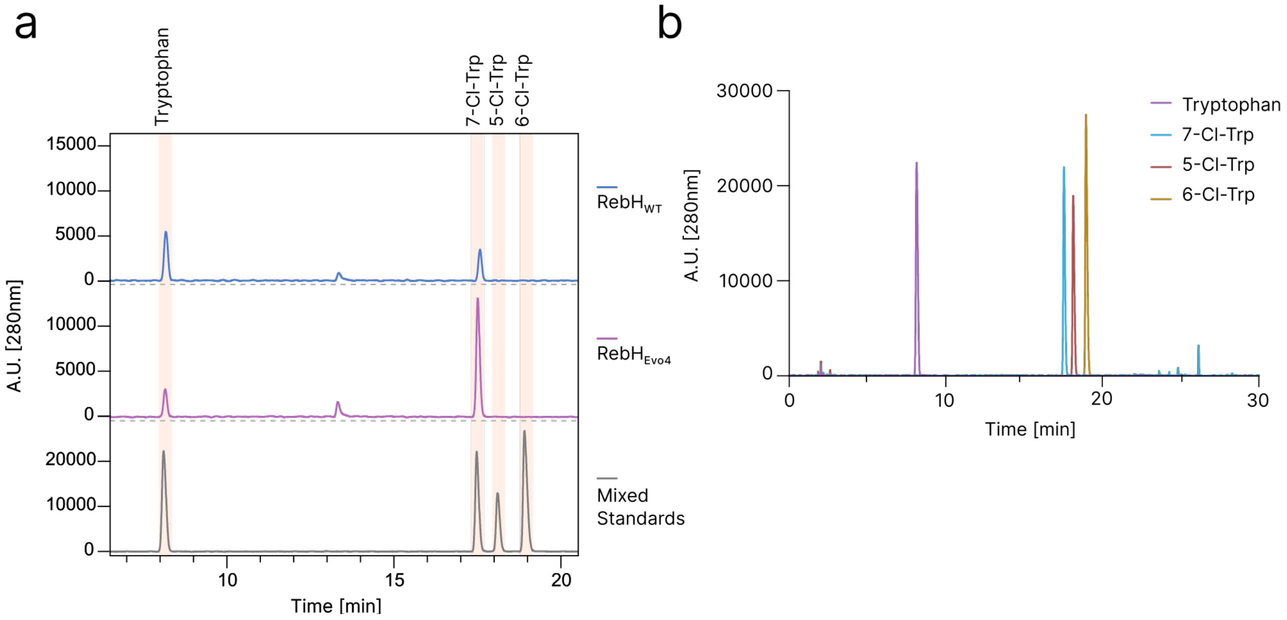

Supplementary Fig. 6 - Confirmation of RebH$_{Evo4}$ regioselectivity

6a. HPLC analysis of products of in vitro chlorination reactions from RebH$_{WT}$ and RebH$_{Evo4}$ confirming both WT and evolved enzymes have exclusive specificity for halogenation at the indole 7 position. Standard mixture included for reference and to confirm separation of regioisomers.

6b. Reference spectra of commercial 5-, 6-, and 7-chlorotryptophan standards run individually to assign peak identity (plotted as one figure).
Source data for this figure is available in the Source Data file 1.

a

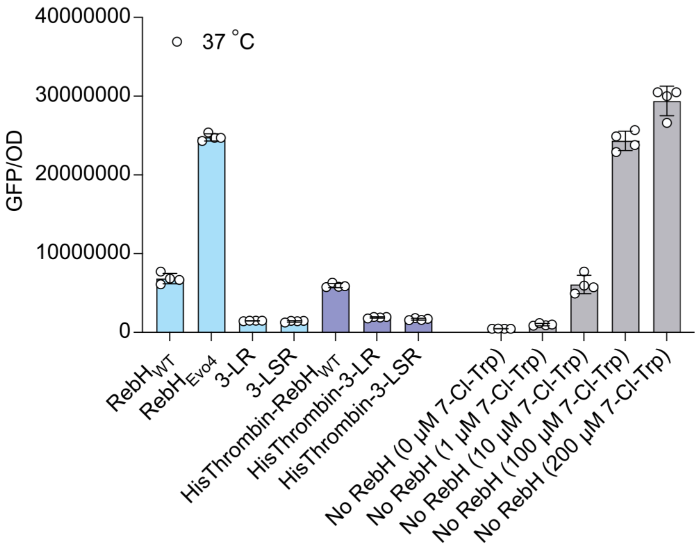

Supplementary Fig. 7 - In vivo benchmarking of $RebH_{Evo4}$

7a. In vivo benchmarking of $RebH_{WT}$ and $RebH_{Evo4}$ against reported mutants with higher in vitro thermostability (3-LR and 3-LSR) using our aaRS biosensor in our Δ*tnaA* strain with overnight sfGFP production as a readout. We tested both transplantation of the 3-LR and 3-LSR mutations into the WT RebH sequence, and expression of 3-LR and 3-LSR combined with its 20 amino acid N-terminal His-Thrombin tag, used in the evolution that produced those mutations[22]. Error bars show mean and standard deviation between 4 biological replicates.
Source data for this figure is available in the Source Data file 1.

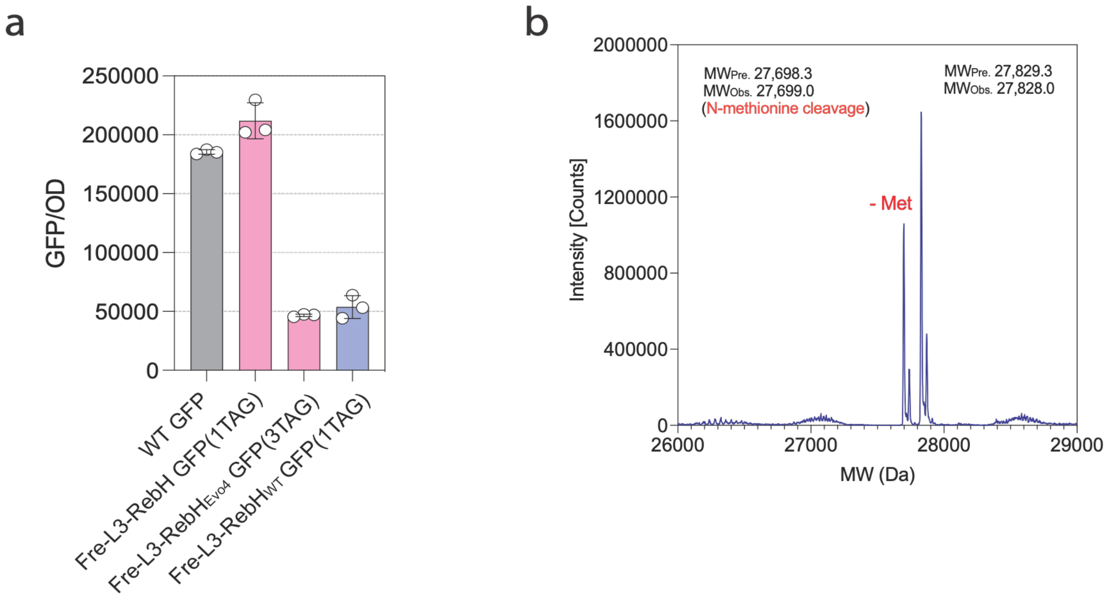

Supplementary Fig. 8 - Amber suppression with autonomous 7-Cl-Trp

8a. Amber suppression of sfGFP using either Fre-L3-RebH$_{Evo4}$ or Fre-L3-RebH$_{WT}$. Amber suppression efficiency is much more efficient when using the evolved enzyme. Error bars show mean and standard deviation between 3 biological replicates.

8b. Intact Mass of sfGFP produced using Fre-L3-RebH$_{Evo4}$ in media supplemented with NaBr. The observed mass matches the expected mass from sfGFP with 7-Br-Trp incorporated at position 151. MWpred. = Predicted molecular weight. MWobs: Observed molecular weight.

Source data for this figure is available in the Source Data file 1.

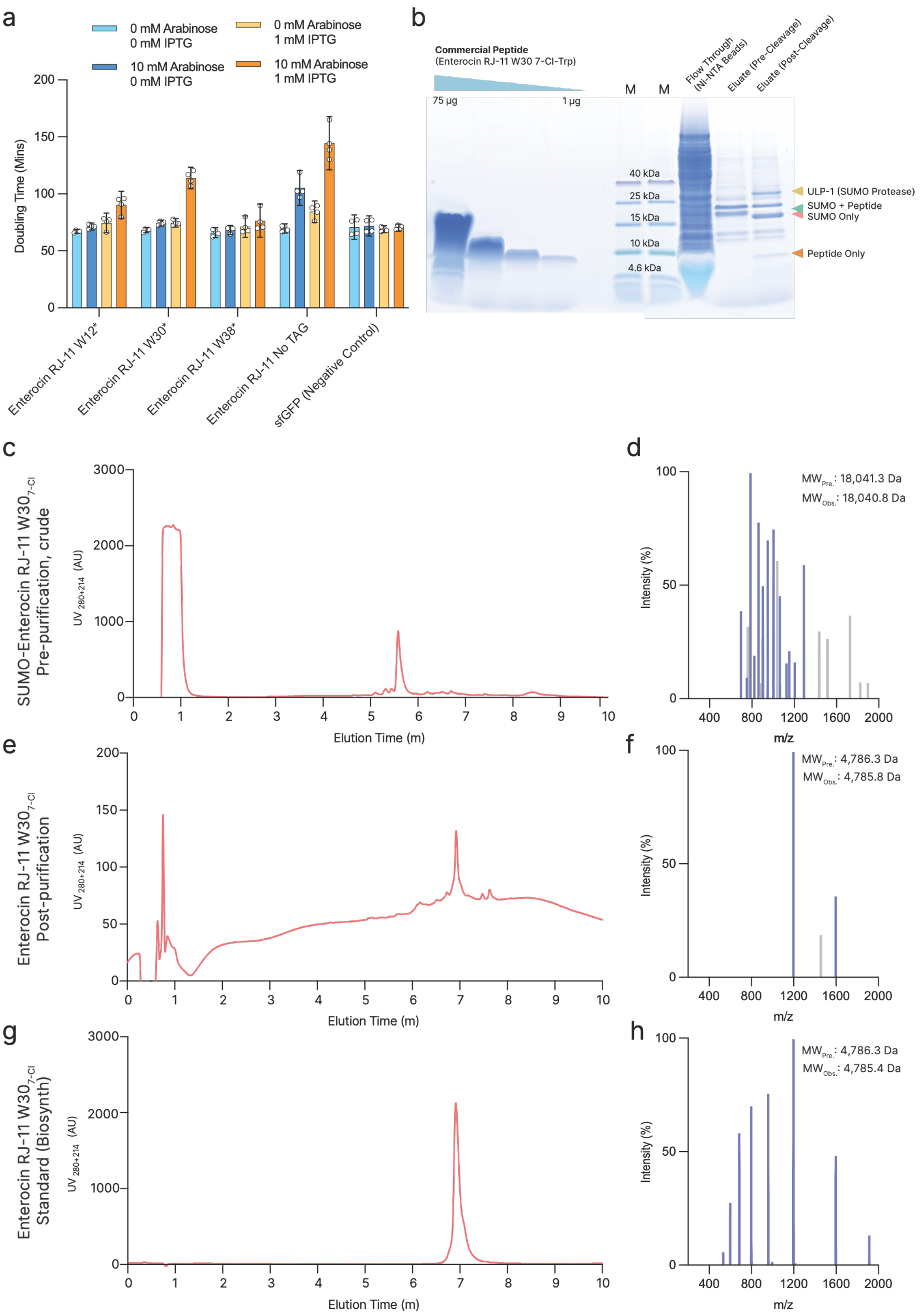

Supplementary Fig. 9 - AMP screening and production

9a. AMP screening data from Fig. 5D presented in bar chart as doubling time of AMP variant-expressing strains with and without AMP and ChPheRS4 expression (Arabinose and IPTG respectively). Data shows the mean and standard deviation of 4 independent biological replicates.

9b. Full SDS-PAGE gel from Fig. 5F with commercially-synthesised peptide for comparison. Image shown is representative of experiments carried out at least twice, on separate days.

9c-9e-9g. Full HPLC traces from Fig. 5G showing crude SUMO-Enterocin RJ-11 $W30_{7\text{-Cl}}$ pre-cleavage and purification, biosynthesised Enterocin RJ-11 $W30_{7\text{-Cl}}$, and commercially synthesised peptide for comparison.

9d-9f-9h. Mass-spectra used to calculate MW values presented in Fig. 5G
Source data for this figure is available in the Source Data file 1.
