# Supplementary material for: Continuous evolution of a halogenase enzyme with improved solubility and activity for sustainable bioproduction
Source: Nat Commun. 2026 Mar 24;17:4357. doi: 10.1038/s41467-026-70981-4 (PMC13171985; doi:10.1038/s41467-026-70981-4)
Supplement: Supplementary file 3 — Description of Additional Supplementary Files [file 41467_2026_70981_MOESM3_ESM.pdf]

Description of Additional Supplementary Files

Supplementary Data 1: Contains the full, annotated plasmid and phage maps for all constructs used in this study, as summarized in Supplementary Table 1.
